# Sex Differences in Spatial Learning and Memory in Valproic Acid Rat Model of Autism: Possible Beneficial Role of Exercise Interventions

**DOI:** 10.3389/fnbeh.2022.869792

**Published:** 2022-04-25

**Authors:** Reza Ghahremani, Reihaneh Mohammadkhani, Iraj Salehi, Seyed Asaad Karimi, Mohammad Zarei

**Affiliations:** ^1^Neurophysiology Research Center, Hamadan University of Medical Sciences, Hamadan, Iran; ^2^Department of Exercise Physiology, Faculty of Sport Sciences, University of Birjand, Birjand, Iran; ^3^Department of Neuroscience, School of Science and Advanced Technologies in Medicine, Hamadan University of Medical Sciences, Hamadan, Iran; ^4^Program in Neurosciences & Mental Health, Hospital for Sick Children, Toronto, Ontario, Canada; ^5^Department of Physiology, University of Toronto, Toronto, Ontario, Canada

**Keywords:** valproic acid, spatial memory, sex difference, exercise, autistic rats

## Abstract

In the current study, we first tried to determine sex differences in spatial learning and memory in the valproic acid (VPA) rat model of autism. Second, the effects of interval training (IT) and continuous training (CT) exercises were examined in male and female offsprings. To induce autism-like animal model, the pregnant rats were injected 500 mg/kg NaVPA (intraperitoneal) at the embryonic day 12.5. IT and CT aerobic exercises were started at postnatal day 56. Then, on postnatal days 84–89, a Morris water maze (MWM) test was conducted on the separate groups of offsprings. Aerobic training was performed on a rodent treadmill with 0% slope for 8 weeks, 5 days/week, and 50 min/day. Unlike control animals, VPA-exposed female offspring had a better performance than VPA-exposed male offspring in MWM acquisition. In the case of MWM reference memory, we did not observe a sex difference between VPA-exposed male and VPA-exposed female offspring. Both IT and CT exercises in both control and VPA-exposed male rats significantly improved MWM acquisition. Moreover, both IT and CT exercises significantly improved MWM acquisition in control female rats. In addition, IT exercise (but not CT) significantly improved MWM acquisition in VPA-exposed female offsprings. Both IT and CT exercises in VPA-exposed that male and female offsprings improved the MWM reference memory. In conclusion, our observation demonstrated that prenatal exposure to VPA affects the spatial learning and memory in a sex dependent manner. We have shown that both IT and CT exercises are able to improve cognitive function in healthy and autistic rat offsprings.

## Introduction

Autism spectrum disorder (ASD) is a prototypic neurodevelopmental disorder that has the following characteristics: deficit in social interaction, language impairment and communication disorder, stereotyped and repetitive behaviors, and sometimes restricted interests and activities (Kientz and Dunn, 1997; American Psychiatric Association, 2013; Ousley and Cermak, 2014). Research has shown that taking antiepileptic drugs, such as valproic acid (VPA) in pregnant women is associated with an increased risk of developing ASD in their children (Rasalam et al., 2005; Bromley et al., 2013; Silvestrin et al., 2013). Studies in rodents have shown that maternal exposure to VPA rises the risk of offspring with autism, producing an animal model of ASD that reflects the various characteristics of patients with ASD (Campolongo et al., 2018; Nicolini and Fahnestock, 2018; Bossu and Roux, 2019). In general, an intraperitoneal administration of VPA into pregnant female rats induces autistic symptoms in the offsprings, and the structures of the brain and the levels of biomarkers in the brain and blood of these offsprings are similar to those of patients with autistic (Christensen et al., 2013; Ornoy et al., 2015).

Finding sex differences in different brain functions looks necessary because the male and female nervous systems respond differently to abnormal physiological conditions (Sickmann et al., 2014). On the other hand, studies have shown that gender may have a significant consequence on cognitive functions in humans (Cahill, 2006; Beggiato et al., 2017) and the behavioral performances of rat (Schneider et al., 2008). Different strategies for decision-making and memory encoding have been reported in men and women.

Men are 4–7 times more likely than women to develop autism (Fombonne, 2009). The male bias in ASD has led to women with ASD being under-researched. Although gender disparity has been reported in ASD, there is little research on gender related to the spatial learning and memory in ASD. It has been shown that the phenotype of women with ASD may be different from that of men (Rivet and Matson, 2011). According to the literature, there are many contradictions regarding the effect of VPA on learning and memory, from “spatial learning and memory enhancement” (Edalatmanesh et al., 2013) to “spatial learning and memory impairments” (Gao et al., 2016; Wu et al., 2018). In the current work, we first tried to determine sex differences in spatial learning and memory in a VPA rat model of autism in the Morris water maze (MWM) test. Second, the effects of interval and continuous exercise training were examined in male and female offspring.

Research has shown that exercise can be used as an effective non-pharmacological strategy to reduce the complications of autism. Exercise increases the cognitive ability, prevents aging-induced failure of memory, protects against neuronal damage, and reduces the symptoms of neurodevelopmental and neuropsychiatric disorders (Cotman et al., 2007; Kim et al., 2010, 2011). Some works have revealed that exercise interventions may be a vital mediator in the treatment of neuropsychiatric, such as autism (Petrus et al., 2008; Lang et al., 2010; Kim et al., 2011).

Treadmill exercise has been shown to improve behavioral outcomes in autistic rats by upregulating the reelin signaling pathway (Seo et al., 2013). However, it is not yet clear what type and intensity of exercise training can have the best effects on ASD. Despite the physiological and clinical relevance of exercise training on autism, no studies have so far provided evidence on the comparisons between the impacts of training regimes of disparate intensities but the same volume on cognition ability. Therefore, in the present study, the effect of interval training (IT) and continuous training (CT) aerobic exercises were examined in male and female VPA-exposed offspring.

## Materials and Methods

### Experimental Animals and the Valproic Acid Rat Model of Autism

**FIGURE 1:**
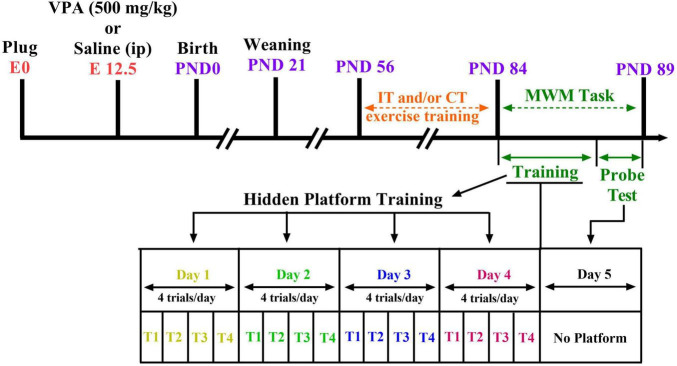
Study design and time diagram of Morris water maze (MWM) task. Two female rats with one male rat were caged overnight and allowed to mate. The first day of pregnancy was determined by the presence of sperm in vaginal smear. On E12.5, valproic acid (VPA)-dams (*n* = 8) received single intraperitoneal (i.p.) injection of NaVPA (500 mg/kg, 3.3 ml/kg); saline-dams (*n* = 7) received a single injection of saline as vehicle (i.p., 3.3 ml/kg). Offsprings were weaned on P21. Interval exercise training (IT) and continuous exercise training (CT) were started at postnatal day 56. Then, on postnatal days 84–89, the MWM test was conducted on the separate groups of pups.

Ethical approval was received by the Animal Study Ethics Committee of our university (Ethics Code: IR.UMSHA.REC.1397.931). In addition, all experiments were done in accordance with the National Institutes of Health Guide for Care and Use of Laboratory Animals. Every effort was made to minimize suffering. Two female Wistar rats mated overnight with an adult male (i.e., 6 weeks of age) of the same strain for pregnancy. The number of impregnated dams was 15. Coition was confirmed at the following morning [on embryonic day 0 (E0)] by the presence of a vaginal plug or sperm in the vaginal smear. Sodium valproate (NaVPA, Sigma) was dissolved in saline to a concentration of 150 mg/ml (to induce a rat model of autism). On E12.5, VPA-dams (*n* = 8) received single intraperitoneal (i.p.) injection of NaVPA (500 mg/kg, 3.3 ml/kg) (Hajisoltani et al., 2019); saline-dams (*n* = 7) received a single injection of saline as vehicle (i.p., 3.3 ml/kg). The rats room temperature was 22 ± 2°C with a light-dark cycle (12 h light–12 h dark), and the animals had free access to tap water and standard laboratory chow. The dams were housed separately and allowed to grow their own litters. On postnatal day 21, the male and female offspring rats were randomly assigned to six groups (*n* = 6–10 in each group): the control group, control plus IT exercise (Control-IT), control plus CT exercise (Control-CT), VPA-exposed animals (VPA), VPA-exposed animals plus IT exercise (VPA-IT), and VPA-exposed animals plus CT exercise (VPA-CT). The total number of animals in all groups was 94 male rats and female rats. Figure 1 shows the experimental design and timetable of the work.

### Training Program

Aerobic training in both interval and continuous exercise groups was performed on a rodent treadmill with 0% slope for 8 weeks, 5 days/week, and 50 min/day (Pereira et al., 2013; Nunes et al., 2015). Before starting the training program from the 8 week old-age of the rats, the animals participated in a treadmill acclimatization period for 5 days, 10–30 min/day at a speed of 10–15 m/min (Nunes et al., 2015). Then, a graded exercise test was performed on the treadmill to achieve maximal running speed in the rats. The initial speed in the test was 10 m/min which was increased by 1 m/min every minute until the rats reached their final speed and could no longer continue the activity; so, the obtained final speed on the treadmill was considered as maximal running speed (Pereira et al., 2013). During the main training sessions, the treadmill speed in the continuous group was a constant load of 70% maximum throughout the training time. The animals in the interval group trained at 1 min high-intensity intervals with 90% maximum speed alternating by 1 min medium-intensity intervals with 50% maximum speed until completing 50 min training time/day (Pereira et al., 2013; Nunes et al., 2015). Thus, the training volume was equal in both groups so that the final results of the research could be compared between them.

### Morris Water Maze Task

#### Apparatus

The Morris water maze (MWM) navigation test, as a hippocampal-dependent test, is widely used to assess spatial learning and memory (Karimi et al., 2017; Habibitabar et al., 2020). Distinguishing between spatial conditions (hidden platform) and non-spatial conditions (visible platform) is the main advantage of the MWM task. In addition, the MWM test environment reduces odor trail interference. The MWM consists of a circular black tank (diameter = 155 cm, height = 60 cm) divided into four equal quadrants and filled with water (35 cm deep and 22 ± 1°C). An invisible platform made of transparent plexiglass (diameter = 10 cm) was placed 2 cm below the water surface in the center of the eastern quadrants (as a target quadrants). A video camera, computer, and tracking software (CCD camera, Panasonic Inc., Japan) were used to record the swim path and performance of rats for further analysis. Large posters prints were used on the wall of the room as visual cues.

#### Habituation

Furthermore, 1 day before the training sessions, the rats swam for 1 min in a tank without a platform to adapt to the MWM test.

#### Hidden Platform Training

The training sessions were conducted according to our previous studies (Karimi et al., 2017; Habibitabar et al., 2020). Briefly, in the training sessions one block of four trials per day was conducted for 4 consecutive days. Each trial started by placing the rat in the middle of one of the four quadrants. Each rat had 90 s swimming time to find the hidden platform. If the rat did not find the hidden platform during this time, it was manually moved to the platform by the experimenter. The time between the two consecutive trials was 10 min. Time to reach the platform (escape latency) and swimming distance during training days were recorded to assess the acquisition of the MWM task. The daily average of all trials from day 1 to 4 was used in our analysis.

#### Spatial Reference Memory (Probe Test or Retention)

The spatial retention test was conducted 24 h after the last training trial and rats swam for 60 s in a tank without platform. The rats were put in the western quadrant (i.e., exactly opposite from where the platform was placed in the training days) and the time spent and distance traveled in target zone was recorded.

#### Visual Test

A visual test was conducted 30 min after the retention test. For this test, the platform was set 1 cm above the water level (in a different zone) and covered with bright color aluminum so that the rat could locate the platform (for 60 s) using a local visual stimulus rather than spatial cues. All tests were conducted between 12:00 and 14:00.

#### Statistical Analysis

GraphPad Prism^®^ 8.0.2 software was used for statistical analysis. Shapiro–Wilk test was used to check the data normality. All data passed normality test (Shapiro–Wilk test was greater than 0.05), so we used one-way and two-way repeated-measures ANOVA analysis followed by a *post hoc* analysis (Newman–Keuls and the Bonferroni multiple comparison tests, respectively). Next, for quantificational evaluation, the area under curve (AUC) was calculated for escape latency and traveled distance, which represented the general cognitive level over 4 consecutive days. The data were presented as mean ± SEM. Differences were considered statistically significant at *p* < 0.05.

## Results

### Sex Difference in the Effects of Prenatal Valproic Acid Exposure on the Morris Water Maze Acquisition in the Male and Female Offspring

**FIGURE 2:**
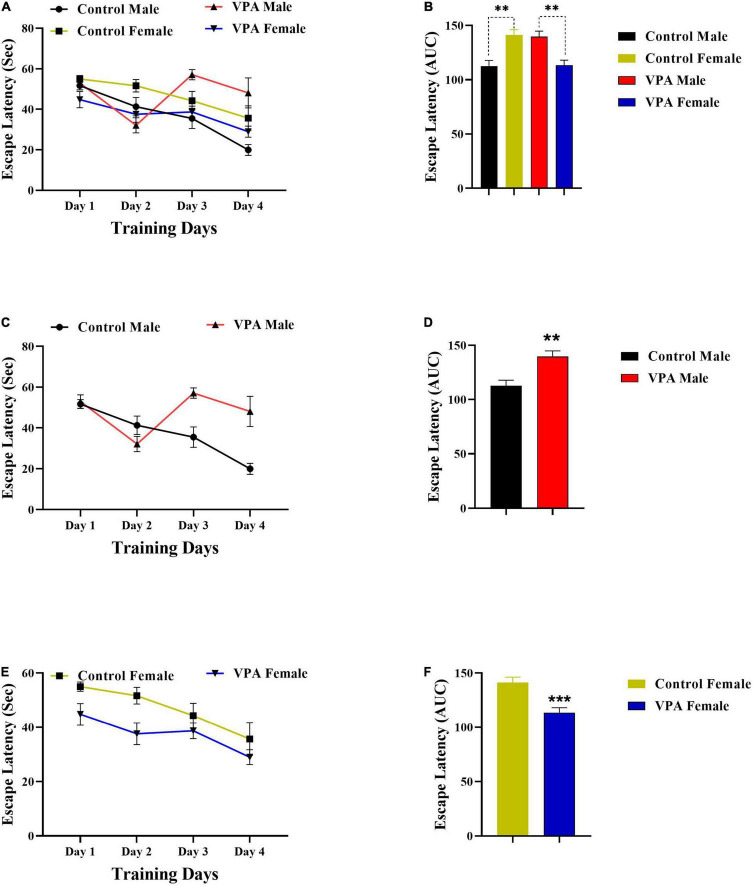
Latency to find a hidden platform during training days. **(A)** Significant sex differences in the escape latency in control and VPA-exposed male and female offspring. **(B)** Area under the curve (AUC) for escape latency. **(C)** Significant differences in the escape latency in control and VPA-exposed male offspring. **(D)** AUC for escape latency in male offsprings. **(E,F)** Same analysis as **(C,D)**, respectively, but for female offsprings. Data are presented as mean ± SEM. **p < 0.01, ***p < 0.001.

**FIGURE 3:**
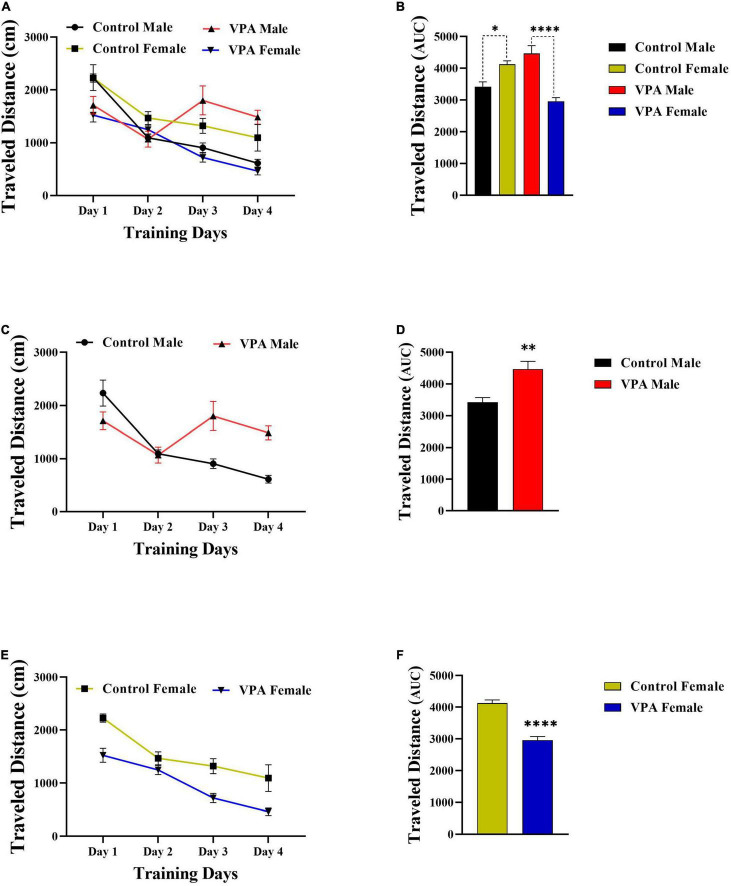
Traveled distance to find the hidden platform during training days. **(A)** Significant sex differences in the distance traveled in control and VPA-exposed male and female offspring. **(B)** AUC for traveled distance. **(C)** Significant differences in the traveled distance in control and VPA-exposed male offspring. **(D)** AUC for traveled distance in male offsprings. **(E,F)** Same analysis as **(C,D)**, respectively, but for female offsprings. Data presented as mean ± SEM. *p < 0.05, ^**^p < 0.01, ^*⁣*⁣**^p < 0.0001.

As there were significant gender differences in the MWM acquisition in control and VPA-exposed male and female offspring (Figures 2A,B, 3A,B), the data were described separately for male and female offsprings. Overall, in MWM task, male control rats outperformed female control rats. But, VPA-exposed female offspring had a better performance than VPA-exposed male offspring.

Valproic acid-exposed male offspring had significantly shorter swim distance and escape latency to find the hidden platform (during training days) than the male control group. Two-way repeated-measures ANOVA revealed significant effect of day effect [*F*(3,53) = 5.476, *p* = 0.0024], significant effect of VPA [*F*(1,53) = 10.56, *p* = 0.0020], and a significant interaction of the two [*F*(3,53) = 3.098, *p* = 0.0344] in escape latency during the training days in male offspring (Figure 2C). Next, the AUC of the escape latency over 4 consecutive days was calculated for each group for statistical comparison. Escape latency to find hidden platform increased in VPA-exposed male offspring animals when compared with male control rats (*p* = 0.0022, Figure 2D). In addition, VPA-exposed male offspring traveled more distance than male control rats {day effect [*F*(2.449,34.28) = 14.30, *p* < 0.0001], VPA effect [*F*(1,14) = 5.528, *p* = 0.0339], and interaction of the two [*F*(3,42) = 9.523, *p* < 0.0001], Two-way RM ANOVA, Figure 3C}. The AUC of traveled distance was calculated for each group. Distance traveled to find the hidden platform increased in VPA-exposed male offspring animals when compared with male control rats (*p* = 0.0027, Figure 3D).

Interestingly, unlike VPA-exposed male offspring, VPA-exposed female offspring had a better performance in MWM training days when compared with the female control group. VPA-exposed female offspring had shorter swimming paths to escape onto the hidden platform, indicating that they had better performance than the female control group. Two-way repeated-measures ANOVA revealed significant effect of day [*F*(3,56) = 5.709, *p* = 0.0018], significant effect of VPA [*F*(1,56) = 12.11, *p* = 0.0010], and a significant interaction of the two [*F*(3,56) = 3.964, *p* = 0.0124] in escape latency during the training days in female offspring (Figure 2E). Next, the AUC of the escape latency over 4 consecutive days was calculated for each group. Escape latency to find the hidden platform decreased in VPA-exposed female offspring animals when compared with female control rats (*p* = 0.0010, Figure 2F). Similarly, VPA-exposed female offspring traveled less distance than female control rats {day effect [*F*(2.100,29.40) = 49.14, *p* < 0.0001], VPA effect [*F*(1,14) = 13.46, *p* = 0.0025], and interaction of the two [*F*(3,42) = 2.548, *p* = 0.0687], two-way RM ANOVA, Figure 3E}. The AUC of traveled distance was calculated for each group. Traveled distance to find the hidden platform decreased in VPA-exposed female offspring animals when compared with female control rats (*p* < 0.0001, Figure 3F).

### Male and Female Performance in the Morris Water Maze Probe Trial Reference Memory

**FIGURE 4:**
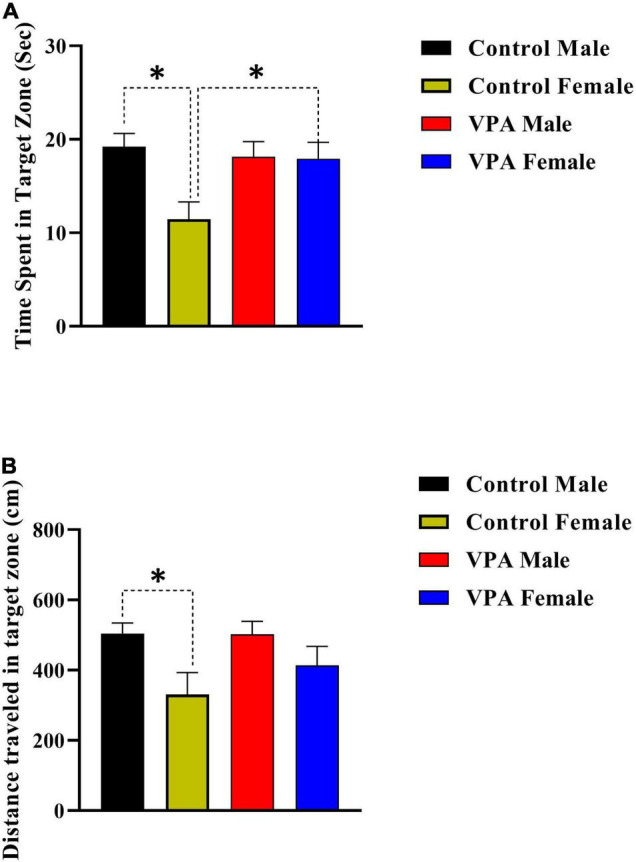
Probe trial performance of male and female rats. There was significant gender difference between male and female control rats but not for VPA-exposed male and female offspring for time spent time **(A)** and traveled distance **(B)** in the target zone. Data are presented as mean ± SEM. *p < 0.05.

The probe trial reference memory test was conducted 24 h after the last training trial. During this test, the platform was removed and time spent and distance traveled in target zone were recorded. There was significant gender difference between male and female control rats but not for VPA-exposed male and female offspring (Figures 4A,B), thus the data were described separately for male and for female offsprings. Control female rats spent less time in the target zone in comparison with control male animals (*p* < 0.05, Figure 4A). However, there was no significant difference between VPA-exposed male and female offspring (*p* > 0.05, Figure 4A). VPA-exposed female offspring animals spent more time in the target zone when compared with female control rats. Time spent in the target zone was almost same for VPA-exposed male offspring and control male animals (Figure 4A).

The data for distance traveled in target zone are shown in Figure 4B. Control female rats traveled less distance in the target zone in comparison with control male animals (Figure 4B). However, there was no significant difference between VPA-exposed male and female offspring (Figure 4B). Moreover, there was no significant difference between VPA-exposed male and female offspring in comparison with male and female control rats, respectively (Figure 4B).

### The Effect of Exercise Interventions on the Morris Water Maze Acquisition in the Control and Valproic Acid-Exposed Male Offspring

**FIGURE 5:**
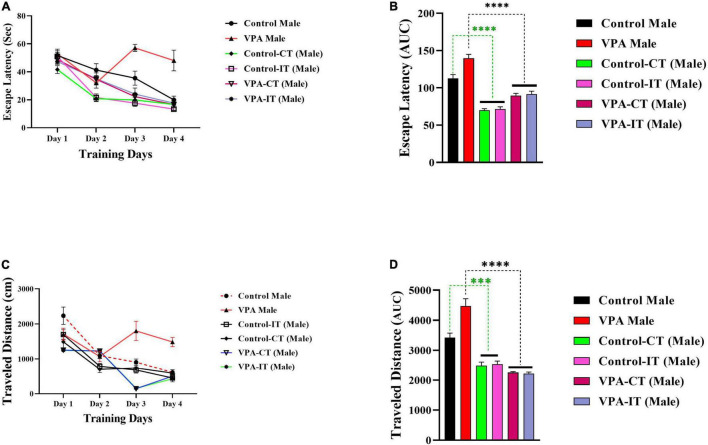
The effects of IT or CT on the MWM acquisition in the control and VPA-exposed male offspring. IT and CT exercise trainings in both control and VPA-exposed male rats significantly reduced the escape latency **(A)** and traveled distance **(C)**. AUC for escape latency **(B)** traveled distance **(D)** in male rats. Data are presented as mean ± SEM. ^***^p < 0.001, ^*⁣*⁣**^p < 0.0001.

The data for the platform location latency and traveled distance in exercise-trained male animals are shown in Figure 5. There were significant differences between experimental groups [escape latency: day effect; *F*(2.822,107.2) = 82.57, *p* < 0.0001, exercise effect: *F*(5,38) = 15.66, *p* < 0.0001, interaction; *F*(15,114) = 7.085, *p* < 0.0001, and traveled distance: day effect; *F*(3,114) = 78.80, *p* < 0.0001, exercise effect: *F*(5,38) = 21.24, *p* < 0.0001, interaction; *F*(15,114) = 9.558, *p* < 0.0001, two-way ANOVA, Figures 5A,C, respectively]. The AUC of the escape latency (Figure 5B) and traveled distance (Figure 5D) was calculated for each group. The Bonferroni multiple comparison test and AUC analysis showed that both IT and CT exercise trainings in both control and VPA-exposed male rats significantly reduced the escape latency and traveled distance.

### The Effect of Exercise Interventions on the Morris Water Maze Acquisition in the Control and Valproic Acid-Exposed Female Offspring

**FIGURE 6:**
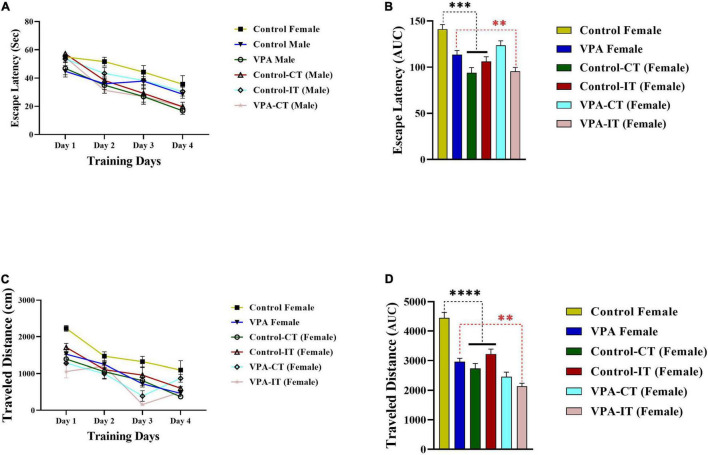
The effects of IT or CT on the MWM acquisition in the control and VPA-exposed female offspring. Both IT and CT exercise trainings significantly reduced the escape latency **(A)** and traveled distance **(C)** in control female rats. IT exercise training (but not CT) reduced the escape latency and traveled distance in VPA-exposed female rats. AUC for escape latency **(B)** traveled distance **(D)** in female rats. Data are presented as mean ± SEM. ^**^p < 0.01, ***p < 0.001, ^*⁣*⁣**^p < 0.0001.

The data for the platform location latency and traveled distance in exercise-trained female animals are shown in Figure 6. There were significant differences between experimental groups [escape latency: day effect; *F*(2.774,110.9) = 84.14, *p* < 0.0001, exercise effect: *F*(5,40) = 3.555, *p* = 0.0094, interaction; *F*(15,120) = 2.248, *p* = 0.0081, and traveled distance: day effect; *F*(2.871,114.8) = 89.31, *p* < 0.0001, exercise effect: *F*(5,40) = 11.51, *p* < 0.0001, interaction; *F*(15,120) = 4.065, *p* < 0.0001, two-way ANOVA, Figures 6A,C, respectively]. The AUC of the escape latency (Figure 6B) and traveled distance (Figure 6D) was calculated for each group. The Bonferroni multiple comparison test and AUC analysis showed that both IT and CT exercise trainings significantly reduced the escape latency and traveled distance in control female rats (*p* < 0.0001). Moreover, IT exercise training (but not CT) significantly reduced the escape latency and traveled distance in VPA-exposed female rats (*p* < 0.01).

### The Effect of Exercise Interventions on the Morris Water Maze Probe Trial Reference Memory in the Control and Valproic Acid-Exposed Male and Female Offspring

**FIGURE 7:**
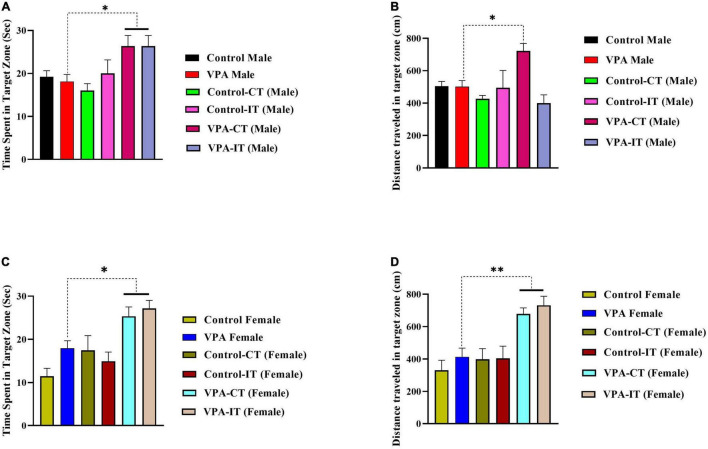
The effects of IT or CT on the MWM probe trial reference memory in the control and VPA-exposed male and female offspring. Both IT and CT exercise trainings in VPA-exposed male rats increased the time spent in the target zone **(A)**. CT exercise training increased distance traveled in the target zone **(B)**. In female rats, that both IT and CT exercise trainings in VPA-exposed male rats increased the time spent **(C)** and traveled distance **(D)** in the target zone. Data are presented as mean ± SEM. *p < 0.05, ^**^p < 0.01, ^*⁣*⁣**^p < 0.0001.

One-way ANOVA followed by Tukey’s *post hoc* analysis for male rats showed that both IT and CT exercise trainings in VPA-exposed male rats significantly increased the time spent in the target zone {[*F*(5,35) = 3.319, *p* = 0.0148], Figure 7A}. In addition, our results showed that CT exercise training (but not IT exercise training) increased distance traveled in the target zone {[*F*(5,35) = 5.328, *p* = 0.0012], Figure 7B}.

In female rats, that both IT and CT exercise trainings in VPA-exposed male rats significantly increased the time spent {[*F*(5,37) = 7.660, *p* < 0.0001], Figure 7C} and traveled distance {[*F*(5,37) = 8.450, *p* < 0.0001], Figure 7D} in the target zone. Additionally, the swimming speed was the same in all rats, which indicates that there is no motor disorder in these animals (data not shown). In addition, in the visual test, all the rats were able to find the non-hidden platform in less than 10 s, indicating that the animals had no visual problems.

## Discussion

A possible beneficial role of exercise interventions and sex differences in spatial learning and memory in prenatal VPA-induced rat model of autism was investigated in the current study. Our observation demonstrated that prenatal exposure to VPA affects the spatial learning and memory in a sex dependent manner. Overall, in MWM acquisition, male control rats outperformed female control rats. However, VPA-exposed female offspring had a better performance than VPA-exposed male offspring. In the case of MWM reference memory, we did not observe a sex difference between VPA-exposed male and female offspring. However, here and in our previous study (Safari et al., 2021), we saw a sex difference between male and female control rats. Both IT and CT exercises in both control and VPA-exposed male rats significantly improved MWM acquisition. Moreover, both IT and CT exercises significantly improved MWM acquisition in control female rats. In addition, IT exercise (but not CT) significantly improved MWM acquisition in VPA-exposed female offsprings. Both IT and CT exercises in VPA-exposed male and female offsprings improved the MWM reference memory.

In our previous work (Safari et al., 2021), we discussed the underlying mechanisms for sex differences in male and female control animals (male control rats outperformed female control rats). However, here we found that VPA-exposed female offspring had a better performance than VPA-exposed male offspring (unlike control animals where male offsprings performed better). Most studies using the VPA model have mainly examined the male sex and sex differences in autistic-like models were described only in few works. Similarly in humans, male and female with autism show different phenotypes. Male mice exposed to VPA showed social impairment and decreased social interaction, which was revealed by the lack of preference for stranger mouse in the three-chamber social experiment (Schneider et al., 2008; Kazlauskas et al., 2019; Ornoy et al., 2019). On the other hand, an increase in repetitive and anxiety-like behaviors has been reported in male and female animals (Ornoy et al., 2019; Sailer et al., 2019). In addition, female animals usually had fewer social and communication problems and fewer repetitive behaviors than male animals (Szatmari et al., 2012; Van Wijngaarden-Cremers et al., 2014; Beggiato et al., 2017). It has been shown that male animals with ASD show poorer perceptual attention to detail (Lai et al., 2012). On the contrary, male animals with ASD performed better in block design performance, while female animals with ASD performed better in the trail making test (Bölte et al., 2011). Edalatmanesh et al. (2013) reported an increase in hippocampal cell density and increased spatial memory in the VPA rat model of autism, but did not examine (or perhaps did not observe) sex differences.

Proposed mechanisms by which VPA exposure during pregnancy causes autistic-like behaviors in both human and rodent offspring are as follow (Ornoy, 2009; Mabunga et al., 2015; Taleb et al., 2021): excitation-inhibition (E:I) imbalance, brain inflammatory challenges, reduction of neurogenesis, oxidative stress, changes in serotonergic, dopaminergic and/or oxytocinergic systems, folic acid deficiency, effects on Wingless and Int-1 (Wnt) signaling, changes in gamma-aminobutyric acid (GABA) and serotonin homeostasis and activity, alteration in neuronal spine density, and changes in calcium/calmodulin-dependent protein kinase II (CaMKII)/protein kinase A (PKA)/protein kinase C (PKC), phosphatidylinositol 3-kinase (PI3K)/protein kinase B (Akt)/mechanistic target of rapamycin (mTOR), glycogen synthase kinase-3β (GSK-3β)/β-catenin signaling pathways. In addition, VPA directly inhibits histone deacetylase (HDAC), causing transient hyperacetylation in the brain (Phiel et al., 2001; Zimmermann et al., 2015).

The number of research has shown that the transcriptional levels of related genes, enzymes, and membrane proteins are likely to be affected by VPA, leading to ASD (Weinstein-Fudim et al., 2019; Taleb et al., 2021). So far, genetic studies identified more than 1,000 genes that contribute to ASD risk (Wiśniowiecka-Kowalnik and Nowakowska, 2019). Behavioral differences and other sex differences in autism may be related to genes that are affected by VPA differently in male and female animals (Weinstein-Fudim et al., 2019). Gender differences in gene expression involved in glutamatergic pathways (such as Unc13a and Cacna1a) may explain sex differences in ASD pathology (Lipstein et al., 2017; Weinstein-Fudim et al., 2019). It is clear that glutamate is involved in learning and memory.

Our present study revealed that both IT and CT exercise trainings improved the spatial learning and memory in the autistic and non-autistic rats. Thus, these results suggest that both CT and IT have beneficial effects on spatial memory. The underlying mechanisms of exercise-induced memory improvement in autistic and healthy rats can be discussed as follows.

Seo et al. (2013) has shown that VPA exposure decreases the expressions of PI3K, p-Akt, and p-ERK1/2 in the hippocampus, and treadmill exercise increases the neurogenesis and the expressions of reelin and its down-stream molecules (PI3K, p-Akt, and p-ERK1/2) in the hippocampus of the autistic rats. They conclude that treadmill exercise improves the spatial memory through the activation of reeling signaling pathway in the VPA-induced autistic rats. In addition, Park et al. (2021) reported that maternal swimming exercise during pregnancy improves memory function by increasing the cell proliferation and inhibiting apoptosis through Wnt/β-catenin signaling cascade activation in the VPA injected pups.

Mice exposed to VPA showed the lower levels of cortical expression of brain-derived neurotrophic factor (BDNF) mRNA (Roullet et al., 2010). It is clear that BDNF is necessary and sufficient for memory (Bekinschtein et al., 2008). Meanwhile, maternal swimming exercise during pregnancy effectively enhanced the BDNF level in the VPA injected pups (Park et al., 2021). Additionally, they showed that maternal swimming exercise inhibited β-catenin, Bcl-2 related X protein (Bax) and cleaved caspase-3 expression and enhanced B-cell lymphoma 2 (Bcl-2) expression in the VPA injected pups (Park et al., 2021).

In addition to the above and based on the literature, the benefits of physical exercise on learning and memory in autistic and healthy rats seem to be mediated by several mechanisms, such as: the production of neurotrophic factors, myelin protection, increased cell proliferation, neurogenesis and plasticity, reduced neuron death, decrease in oxidative stress, and neuroinflammation (Phillips et al., 2014).

Some neurodevelopmental disorders, such as autism, are quite limited to humans. Besides, there are many limitations and precautions in designing rodent models. The most important one is that animals cannot replicate all of the uniquely human components of autism. In conclusion, our observation demonstrated that prenatal exposure to VPA affects the spatial learning and memory in a sex dependent manner. We have shown that both IT and CT exercises are able to improve the cognitive function in healthy and autistic rat pups. Since the nervous system controls the cognitive behavior, these sex-related functional differences may be associated to the sex-specific structure of the neuronal circuits in the nervous system (Cosgrove et al., 2007). Future studies are needed to validate our findings in humans ASD, such as behavioral testing across sex.

## Data Availability Statement

The original contributions presented in the study are included in the article/supplementary material, further inquiries can be directed to the corresponding author.

## Ethics Statement

The animal study was reviewed and approved by the Animal Study Ethics Committee of Hamadan University of Medical Sciences.

## Author Contributions

SK designed the project, wrote the manuscript, performed the statistical analysis, revised the manuscript, and supervised the project. RG, RM, and IS were involved in laboratory works and experimental design of the work. MZ was involved in laboratory assessments. All authors read and approved the final results.

## Conflict of Interest

The authors declare that the research was conducted in the absence of any commercial or financial relationships that could be construed as a potential conflict of interest.

## Publisher’s Note

All claims expressed in this article are solely those of the authors and do not necessarily represent those of their affiliated organizations, or those of the publisher, the editors and the reviewers. Any product that may be evaluated in this article, or claim that may be made by its manufacturer, is not guaranteed or endorsed by the publisher.
